# Review of History of Basic Principles of Burn Wound Management

**DOI:** 10.3390/medicina58030400

**Published:** 2022-03-07

**Authors:** Hyunjin Kim, Seongmee Shin, Donghoon Han

**Affiliations:** 1 Department of Plastic Surgery, Hangang Sacred Heart Hospital, Hallym University College of Medicine, Seoul 07247, Korea; msgod456@naver.com; 2 Department of Internal Medicine, Kangnam Sacred Heart Hospital, Hallym University College of Medicine, Seoul 07441, Korea; 93tjdal@naver.com

**Keywords:** burn management, antibiotics, fluid resuscitation, history, burn wound care

## Abstract

Thermal energy is an essential and useful resource to humans in modern society. However, a consequence of using heat carelessly is burns. Burn injuries have various causes, such as exposure to flame, radiation, electrical, and chemical sources. In this study, we reviewed the history of burn wound care while focusing on the basic principles of burn management. Through this review, we highlight the need for careful monitoring and customization when treating burn victims at each step of wound care, as their individual needs may differ. We also propose that future research should focus on nanotechnology-based skin grafts, as this is a promising area for further improvement in wound care.

## 1. Introduction

Thermal energy is essential for human life in modern society. However, a consequence of using thermal energy is burns [1]. The skin is made of proteins and acts as a barrier, which protects the organism from the outside environment, regulates body temperature, and prevents fluid loss [1,2]. In a burn injury, this protective barrier is damaged and proper treatment should be provided immediately. Although there are various types of burn injuries, including those due to flame, radiation, electrical, and chemical agents, the goal of all burn management is the same: stop the burning process, minimize scarring, relieve pain, reduce secondary infection, and prevent future complications, such as dysfunction of the injured site or burn shock [3,4]. Early emergency treatment, including aggressive surgical excision, skin regeneration, and pain control, as well as psychological support, should be considered to improve burn management [5,6,7,8]. In this paper, we review the history of burn wound care, focusing on the basic principles of burn management.

## 2. Medical Approaches

### 2.1. Fluid Resuscitation

**Figure 1:**
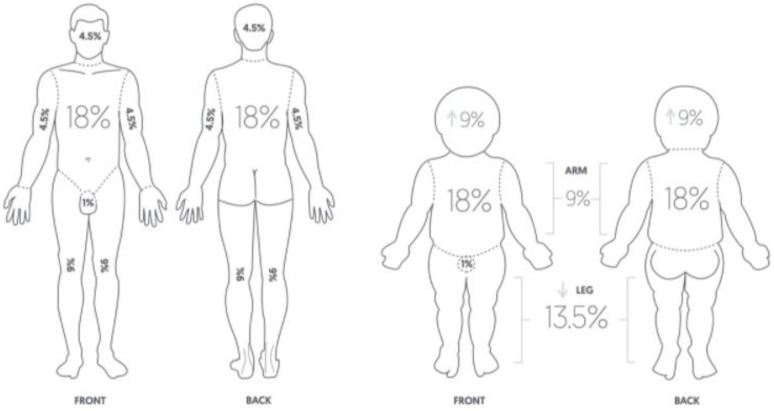
Wallace “rule of nines” for adults and children [17]. With permission from Pulsenotes.

Rapid and adequate intravascular volume supplementation is a cornerstone of the prevention of burn shock [9]. Delayed or inadequate fluid replacement can result in hypovolemia, leading to tissue hypoperfusion, hypovolemic shock, and multiple organ failure [10]. In the 1940s, there were large urban fires, such as the one at the Coconut Grove nightclub (Boston, MA, USA), and physicians found that some patients survived large burns but died from secondary shock [11,12]. Thus began fluid resuscitation studies in severely burned patients. In 1942, Cope and Moore introduced the concept of fluid resuscitation in burn victims, being tailored to individual patient needs [13]. Evans developed a burn surface area–weight formula for the demand of fluid replacement in patients with burns in 1952 [14]. Wallace published the rule of nines, a tool used to assess the total body surface area (TBSA) in patients with high-degree burns as a quick and easy assessment tool to ascertain the severity of the burns and intravenous fluid needs (Figure 1) [15,16].

The rule of nines can be modified based on body mass index and age [18,19]. In 1968, Baxter and Shires formulated a measure of fluid volume requirement by weight and percentage of the TBSA, known as the Parkland formula [20,21]. During the 1960s, researchers at the Brooke and Parkland hospitals in Texas developed formulas that only used lactated Ringer’s (LR) solution, and no plasma, for the first 24 h following burn injury [21]. The modified Brooke formula (which estimates the first 24 h volume as LR solution, 2 mL/kg/total body surface area burned (TBSA), with half of this delivered over the first 8 h) and the Parkland formula (similar to the Brooke formula but 4 mL/kg/TBSA) are the most commonly used formulas for resuscitation of adult burn patients today [22]. Albumin replaced plasma in most resuscitation regimens and was primarily used during the second 24 h, at a dose of 0.3 to 0.5 mL/kg/TBSA for the day [22]. However, recent data suggest that the formula does not accurately predict fluid requirements in patients with large burns and that patients recently treated using the formula frequently required higher volumes of fluid than was predicted [23,24]. Blumetti et al. reported that only 14% of adequately resuscitated and 12% of over-resuscitated patients met the Parkland formula criteria in a retrospective study of patients resuscitated using the formula over a 15-year period [25]. Daniel et al. reported that a restrictive fluid regimen showed a higher survival rate than the liberal Parkland regimen based on data from the German burn registry [26]. Fluid resuscitation formulae are only guides to assist in the estimation of fluid requirements. Therefore, the volume of fluid should be customized for each patient according to the extent of burn injury and patient condition [27]. Regardless of the formula or strategy used, frequent adjustments based on clinical indicators of the adequacy of resuscitation are required in the first 24–48 h (Table 1) [27].

### 2.2. Nutrition for Wound Healing

Providing adequate nutrition is important for burn wound healing and recovery [29,30]. Aggressive feeding in children is associated with decreased muscle protein catabolism, a reduced rate of burn sepsis, and lower bacterial counts from excised tissue [31]. Similarly, in adults, early nutritional support is associated with shorter hospital stays, faster wound healing, and reduced risk of infection [32]. However, several nutritional factors must be carefully considered. Excess carbohydrate consumption can lead to hyperglycemia, which can aggravate systemic inflammation and muscle degradation [33,34,35]. Excess fat supply may exaggerate the immunosuppressed state, and since major burn injuries may also result in immunosuppression, this result may increase the risk of infection and sepsis [30,36,37]. Proteins play an essential role in every step of the wound-healing process. Proteins are necessary for collagen synthesis, angiogenesis, fibroblast proliferation, immune function, tissue remodeling, wound contraction, and skin structural proteins [30,38]. Moreover, leukocytes, monocytes, lymphocytes, and macrophages require protein for their formation and function in mounting an immune response. Protein deficiency results in impaired fibroblast proliferation and collagen synthesis during the proliferative phase of healing [39,40].

### 2.3. Control of Infection in Burns

Wound infection, including sepsis, is the most serious complication of burns in the acute period following burn injury [41,42]. Approximately 73–85% of all deaths that occur within the first 5 days of injury are due to sepsis [43,44,45]. Colebrook first introduced the concept of safeguarding burn wounds from infection. He suggested that burn wounds could easily be infected with bacteria; thus, strict infection control could help prevent wound infection by reducing the transfer of organisms among patients in a burn center [46,47].

When administering systemic antibacterial agents to burn patients, clinicians should carefully consider the emergence of antibiotic-resistant organisms [48]. Coban provided two suggestions to overcome infection by resistant microorganisms: (1) the burn center should maintain quality control against microorganisms, and antibiotic agent administration should consider the antibiotic resistance trends within each burn center; (2) systemic antibiotics should only be administered for a short period to patients with burns [49]. Prophylactic use of antibiotics enables the development of secondary infections (commonly diarrhea); therefore, it should not be applied routinely in all burn cases [49]. Prophylactic use is recommended only during the immediate perioperative period surrounding excision or grafting of the burn wound when there is an increased risk of bacteremia. Antibiotic therapy should be started immediately before the procedure and should generally be discontinued within 24 h [48]. Most importantly, clinicians must try to prevent burn wound infection by monitoring alterations in the wound character, odor, or amount of pus drainage at the time of each dressing change, and by practicing aseptic dressing techniques, particularly when handling the open wound and dressing materials. In addition, the frequency of dressing should be based on the wound condition [48].

## 3. Surgical Approaches

### 3.1. Burn Wound Dressing

Wound dressings and healing agents are routinely used to treat burn wounds [50]. Burn dressings can protect the skin from infection and further skin damage, promote re-epithelialization, and decrease pain [11,51]. Humidity and heat-preserving dressings, as well as moist dressings, are recommended for burns [52]. An ideal dressing that provides a moist and humid environment for wound healing has not been identified thus far [52,53,54]. Small burns can be effectively treated with simple dressings, but more careful management is necessary for larger burns to reduce body fluid loss caused by damage to the skin barrier [55]. 

In 1500, Pare treated burn wounds with onion. This is the first description of burn wound management using dressings [51]. In 1797, Kentish reported that pressure dressings could alleviate burn pain and blisters. In 1839, Dupuytren reviewed treatment with occlusive dressings and developed a classification of burn depth that is still widely used [56,57,58].

**Figure 2:**
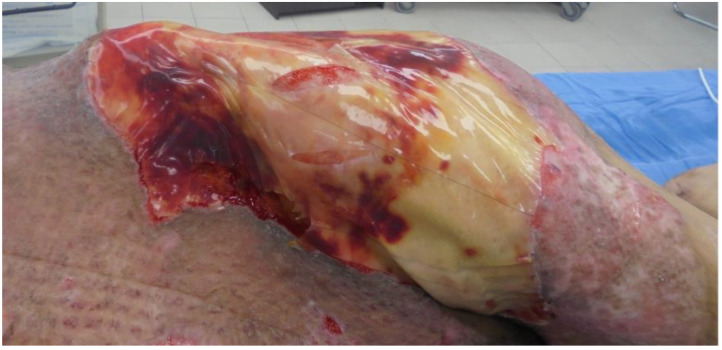
Integra applied on burn wound.

More recently used dressings for burn wounds can be divided into four categories: (i) Biological dressings, including allograft, xenograft, and human amnion. These dressings effectively promote the healing of wounds for further skin grafting; however, they cannot replace permanent skin and are associated with inconsistent quality, limited supply, and increased risk of bacterial pathogen transfer [11,59,60]. (ii) Conventional dressings use Vaseline gauze or silicone sheets. Although these dressings are widely used, they tend to stick to the wound surface, which can damage the newly epithelialized surface and delay wound healing [51,61]. Nevertheless, Lucattelli et al. reported that applying silicone gel was particularly effective in re-epithelialization despite physically and biologically interacting with injured tissue [62]. In addition, Xeroform gauze is useful for superficial, partial-thickness burns and split-thickness skin graft donor sites [63,64,65]. Xeroform consists of a mixture of petroleum jelly and bismuth tribromophenate. Petroleum jelly of Xeroform creates an occlusive, nonadhesive barrier that helps the wound to remain moist, and bismuth tribromophenate has an antimicrobial effect [64,66]. (iii) Biosynthetic dressings use a material functionally similar to skin [11]. Biobrane™ is a biosynthetic dressing recommended for superficial, partial-thickness burns [67]. It is made from a porcine dermal collagen-bonded nylon membrane on silicon scaffolding. The collagen component initially adheres to the fibrin on a clean wound surface, and this adherence contributes to pain reduction. The silicone outer layer can prevent excessive water loss, thereby promoting a desirable moist environment for wound healing. Its transparency also allows for inspection to assess the wound condition [68]. However, nowadays, this material is not routinely used. The most widely used dermal regeneration template is Integra (Integra LifeScience Corporation, Plainsboro, NJ, USA), which is a bilayer composed of a matrix of bovine collagen cross-linked with glycosaminoglycans from shark chondroitin sulfate with an overlying protective silicone layer [69]. The use of Integra templates in reconstructive surgery has been described in burns, scalp, limbs, abdominal wall, degloving injuries, keloids, hypertrophic scars, diabetic foot ulcers, and necrotizing soft-tissue infections among other uses (Figure 2) [70,71,72,73,74,75,76,77]. Although Integra has been shown to be an effective reconstructive tool with excellent functional outcomes, aesthetic results, and high rates of long-term engraftment, infection associated with Integra use was the most common complication [78,79,80,81,82]. (iv) Antimicrobial dressings are widely used in burn management to prevent wound infection. Antimicrobial dressings use products containing silver, nanocrystalline silver, iodine (cadexomer or povidone iodine), or honey and mafenide acetate, all of which can prevent bacterial colonization [11,83,84,85,86,87]. The usefulness and effect of silver for wound treatment has been known since 69 BC [88]. Silver sulfadiazine is widely used for the management of second-degree burns, but it has poor outcomes in burn wound care, particularly with respect to infection and epithelialization [89,90,91]. Nanocrystalline silver dressings were developed and introduced in the late 1990s and are the latest forms of silver-based wound dressings. These products were developed to overcome some of the weaknesses of earlier silver dressings [92]. Dressings with nanocrystalline silver are superior to silver sulfadiazine and silver-free dressings for burns in terms of epithelialization, infection, and pain control [93,94,95,96].

Iodine is a well-known antiseptic agent that is widely used in wound dressing [97]. Povidone iodine does not cause a delay in wound healing or have harmful effects in patients with burns [98,99,100]. Cadexomer iodine is a slow-release antimicrobial agent that can absorb excess wound exudates while maintaining a sustained level of iodine in the wound. Although studies on the effect of cadexomer iodine in patients with burns is lacking, it is effective against antibiotic-resistant bacteria [101]. 

Honey has antibacterial properties and enhances tissue growth [102,103]. Several studies have revealed that dressing with honey had a better outcome than dressing with silver [104,105,106,107].

Mafenide acetate (Sulfamylon) is a widely used antimicrobial agent to prevent bacterial infection on a variety of burn wounds, such as full-thickness burns and burns with eschar, post-excision, and autograft [87,108,109,110,111]. Sulfamylon inhibits the nucleotide synthesis of bacteria, and it has bacteriostatic effects on Gram-positive and Gram-negative organisms [112,113]. Sulfamylon was used in a 10% topical water-soluble cream in 1964 [114]. However, the high osmolality of 10% Sulfamylon cream was associated with neoeschar formation and wound pain [112,115]. Several animal experiments and clinical studies demonstrated that the use of 5% Sulfamylon solution dressing provided a better result with less side effects [116,117,118]. Finally, the 5% Sulfamylon became widely used for burn wound treatment [108].

### 3.2. Surgical Treatment of Burns

Surgical treatment of burns includes two procedures, namely, skin excision and skin grafting. In 1607, Hildanus mentioned that the removal of burn eschars could facilitate drainage of serous fluid and allow for better medication penetration [119]. Young, McCorkle, and Silvani, and Saltonstall and Lee reported successful experiences with surgical excision and skin grafting in patients with deep burn wounds [120,121,122]. In the 1970s, Janzekovie introduced tangential excision, in which early excision of burns resulted in better prognosis [123]. Subsequently, Monafo performed tangential excision and grafting for the treatment of patients with larger burns [124]. In a randomized, prospective study, Engrav et al. reported that early tangential excision and grafting of deep second-degree burns improved outcomes, such as reduced mortality and hospitalization duration, compared to conservative treatment methods [125]. Tompkins et al. reported that prompt eschar excision reduced mortality in a retrospective study from 1974 to 1984 [126]. Saaiq et al. also reported that early excision and grafting had better outcomes in terms of graft take, post-graft hospitalization, and mortality, in patients with deep burns covering up to 40% of the TBSA [127]. 

Early excision is defined as excision of the entire burn wound within 24 h to approximately 7 days [128,129,130,131]. A recent meta-analysis found that early excision of burns was beneficial in reducing mortality in patients without inhalational injury [130]. However, the optimal timing for early excision still remains controversial. Moussa et al. reported that early excision, within 24 h, resulted in better outcomes, but that delayed excision, up to 72 h after the burn injury, might be reasonable in selected patients [132].

Negative pressure wound therapy (NPWT), including topical negative pressure therapy and vacuum-assisted closure, has been used in the context of open-wound management; the interface foam is applied directly to the wound bed, which is visible at the body surface [133,134]. NPWT has been applied to various surgical wounds since 1997 [135]. It can accelerate wound healing on both acute and chronic wounds, and it has been widely used on wounds with soft tissue defects [136,137]. Although the mechanism of NPWT promoting wound healing is not fully understood, some researchers suggest that it may contribute to maintaining a moist environment for wounds, removing inflammatory exudate from the wound, and reducing exposure to pathogens [138,139]. NPWT improved the outcomes of various aspects of burn wound care, including acute burns, autografts, skin grafts, donor sites, and large burns [140,141,142,143,144]. Although further research is needed to explore the clinical application of NPWT for burns, it has proven to be a beneficial aspect in various aspects of burn wound care.

Skin grafts may be surgically attached to the burn site to promote healing. Two types of grafts, autografts and allografts, can be used to cover the wound bed [145]. Girdner reported the first successful use of allografts in severe and extensive burn wounds in 1881 [146]. In 1954, Jackson introduced a combined grafting technique using narrow strips of allograft and autograft in a granulating or excised wound [147]. Alexander et al. developed a simple technique, which applied a widely meshed skin autograft and then covered it with allogenic skin [148].

Despite their imperfections, existing dressings and tissue-engineered skin substitutes have significantly improved clinical outcomes for burn victims, leading to increased survival rates and improved quality of life [61,149,150]. Stem cells derived from various sources have been used for their regenerative properties [151,152]. However, further research is needed for the use of stem cells in burn management. Nowadays, cells, biomaterial, and delivery-based materials are routinely used when designing skin substitutes [151,153,154]. Furthermore, emerging nano-based therapeutic systems, such as nanoparticles (nonpolymeric and polymeric) and scaffolds (nanofibers, films and membranes, foams and sponges, and hydrogels), are also being used in burn-wound-healing processes. Nanomedicine shows great potential to improve and enhance the healing process in burn wounds. However, future research on nanomedicine, along with toxicology and safety assessments, will be necessary for further development [155]. 

## 4. Conclusions

In modern society, thermal energy is an essential resource; however, its use increases the risk of sustaining burns. Evidence-based treatment plans can improve the survival of patients with burns and lead to better prognosis. Many advanced techniques have been developed for burn wound care, and several materials have been developed to reconstruct burnt skin and treat deformities. In the future, active research on skin recovery should be conducted to improve the management of burn wounds.

## Figures and Tables

**Table 1:** Summary of Adult Formulas for burn fluid replacement [28].

Formula	Crystalloid	Colloid	Glucose	Instructions for Administration
**Cope and Moore**	75 mL/%TBSA burn oral electrolyte replacement solution	75 mL/%TBSA burn FFP	2000 mL fruit juice PO or 2000 mL 5% dextrose IV	Half over the first 8 h, half over the second 16 h
**Evans**	1 mL/kg/%TBSA burn of NS	1 mL/kg/%TBSA burn FFP	2000 mL 5% dextrose	Half over the first 8 h, half over the second 16 h
**Brooke**	1.5 mL/kg/%TBSA burn of LR	0.5 mL/kg/%TBSA burn FFP	2000 mL 5% dextrose	Half over first 8 h, half over second 16 h
**Parkland**	4 mL/kg/%TBSA burn of LR	None	None	Half over the first 8 h, half over the second 16 h
**Modifed Brooke**	2 mL/kg/%TBSA burn of LR	None	None	Half over the first 8 h, half over the second 16 h

Abbreviations: %TBSA, percent total body surface area; NS, normal saline; LR, lactated Ringer’s; FFP, fresh frozen plasma.

## Data Availability

All information included in this review is documented by relevant references.
